# Powered knee and ankle prostheses enable natural ambulation on level ground and stairs for individuals with bilateral above-knee amputation: a case study

**DOI:** 10.1038/s41598-022-19701-8

**Published:** 2022-09-14

**Authors:** Sarah Hood, Suzi Creveling, Lukas Gabert, Minh Tran, Tommaso Lenzi

**Affiliations:** Department of Mechanical Engineering and Utah Robotics Center, University of Utah, Salt Lake City, UT 84112 USA

**Keywords:** Biomedical engineering, Mechanical engineering, Translational research

## Abstract

Ambulation with existing prostheses is extremely difficult for individuals with bilateral above-knee amputations. Commonly prescribed prostheses are passive devices that cannot replace the biomechanical functions of the missing biological legs. As a result, most individuals with bilateral above-knee amputations can only walk for short distances, have a high risk of falling, and are unable to ascend stairs with a natural gait pattern. Powered prostheses have the potential to address this issue by replicating the movements of the biological leg. Previous studies with individuals with bilateral above-knee amputations have shown that walking with powered prostheses is possible. However, stair ambulation requires different kinematics, kinetics, and power than walking. Therefore, it is not known whether powered prostheses can restore natural ambulation on stairs for bilateral above knee individuals. Here we show a case study with an individual with bilateral above-knee amputations using a pair of lightweight powered knee and ankle prostheses for walking and stair ambulation. The kinematic analysis shows that powered prostheses can restore natural leg movements, enabling the individual to walk and climb stairs using different gait patterns, such as step-over-step or step-by-step, one step or two steps at a time. The kinetic analysis shows that the powered prostheses can restore natural ankle push-off in walking and positive knee power generation in stair ascent, which are fundamental biomechanical functions of the missing biological legs. This case study is a first step towards enhancing functional mobility and quality of life for individuals with bilateral above-knee amputations through powered knee and ankle prostheses.

## Introduction

Performing common activities of daily living with bilateral above-knee amputations is difficult^1^. Existing knee prostheses are mechanically passive devices that cannot actively generate movements^2^. Thus, during ambulation, users must inject all the energy necessary to propel the prostheses movements through their residual limbs. These compensatory movements result in an unnatural and inefficient gait pattern^3,4^. As a result, individuals with bilateral above-knee amputations can only walk for short distances and have a high risk of falling^4,5^. Most importantly, climbing stairs requires individuals to use their upper body strength, basically pulling themselves up the stairs because the knee prostheses cannot generate extension torque as necessary to lift the body up the step^6,7^. Most individuals do not have the strength to perform this movement, as a result, they are unable to ascend stairs^7^.

During walking, individuals with bilateral amputations can use the movement of their residual limbs/hips to control the prostheses movements^8,9^. In late stance, the ground reaction force and center of pressure move forward towards the prosthetic toe^9^, generating a flexion moment around the prosthetic knee joint. This flexion moment causes the knee to flex in early swing as necessary to clear the ground and avoid tripping. Also, in early swing, individuals perform quick hip flexion movement generating the momentum necessary for the prosthetic knee joint to start extending^9,10^. This knee extension movement brings the foot forward in preparation for the next step to be taken. At the end of swing, individuals quickly extend their hips to generate a moment that forces the extension movement of the prosthetic knee to stop so that when they begin to load their body weight on the prostheses the knee joint is fully extended^9,10^. Microprocessor-controlled knee prostheses can lock the joint in stance, preventing the knee from collapsing as the user loads it^11,12^. During early stance, individuals perform a strong hip extension, which keeps the prosthetic knee fully extended and propels the body forward. These unnatural compensatory movements make walking highly inefficient and unstable^3,4,13^.

Powered prostheses present a possible solution to restoring natural gait patterns during walking. Using motors, sensors, and control powered prostheses can actively generate movements imitating the biomechanical function of the missing biological leg^14^. A previous study with one individual with bilateral above-knee amputation has shown that paired research powered knee and ankle prostheses can approximate the joint kinematics of the biological leg during walking^15^. The study participant could walk with adequate clearance during swing and showed controlled weight acceptance during stance. In this study, the two powered knee and ankle prostheses were explicitly synchronized using inter-prosthesis communication and the authors claim that the synchronization was necessary to ensure proper function, for example by avoiding the two prostheses to transition to swing at the same time^15^. Another case study was performed with an individual with bilateral above-knee amputation comparing walking with commercially available microprocessor-controlled and powered prostheses^16^. Specifically, this study used the second generation of the Ossur Power Knee combined with the first generation of the BiOM from iWalk, which was later acquired by Ottobock and re-branded as Empower. The case study showed that when using the commercially available powered knee and ankle prostheses there is an increase in step length symmetry, decreased vertical ground reaction force, and increased limb transition work during walking compared to the microprocessor prostheses^16^. Thus, powered prostheses have shown successful walking for individuals with bilateral above-knee amputations^15,16^, although the results do not show significant improvements in clinical outcomes.

Ascending stairs with microprocessor-controlled prostheses is much more challenging than walking on level ground^6,7^. The compensatory movements performed by an individual with bilateral above-knee amputations in walking are not enough during stair ascent. Due to the fundamental limitations of exiting prostheses, individuals with above knee amputation ascend stairs with a substantially different gait pattern than nonamputees. During swing, the prosthetic knee cannot actively flex as necessary to clear the step. Thus, individuals must move their hips in a circular motion, a movement commonly known as hip circumduction, to clear the steps and place the foot on the step in front of them. Different from nonamputees, the prosthetic foot is not placed flat on the step and the prosthetic knee is not flexed. Rather the foot is placed on the edge of the step and the knee is fully extended. To actually climb the step, individuals must keep the prosthetic knee fully extended which requires starting weight acceptance with the hip unnaturally flexed. Then they can pull themselves up and over their prostheses with their arms while extending their hips to keep the prosthetic knee fully extended and in contact with the step. These compensatory movements require significant exertion and the risk of falling is high as the prosthetic foot placed on the edge of the step may slip, causing the user to fall. As a result, most individuals with bilateral above-knee amputations cannot climb stairs.

Powered prostheses can replicate the biomechanical function of the missing biological leg^14^. Active control of powered prosthetic joint movements allows for proper toe clearance as well as a natural placement of the foot on the step^17^. Moreover, powered prostheses can provide active knee extension torque in stance, which allows for the step to be climbed without using the upper body^18^. Different control strategies have been used to achieve stair ascent with the natural step-over-step gait pattern. Initial efforts required fine tuning the powered prostheses controllers to the specific step height and only allowed for a fixed cadence^19–21^. More recently, a controller has been shown that enables users to climb stairs of different heights and at their preferred cadence^18^. This new controller has also shown the ability to adapt online to user’s preferred gait pattern (i.e., step-by-step, step-over-step, and two steps at the time). Powered knee and ankle prostheses can enable individuals with unilateral above-knee amputation to climb stairs with a natural step-over-step gait pattern.

Despite the success with unilateral above-knee users, to the best of our knowledge, stair climbing with powered knee and ankle prostheses has never been shown for individuals with bilateral above-knee amputations. The goal of this case study is to assess whether powered knee and ankle prostheses can restore natural gait patterns both on level ground and on stairs for individuals with bilateral above-knee amputations. To this end, we fit one study participant with bilateral above-knee amputations with two research lightweight powered knee and ankle prostheses. The powered prostheses use ambulation controllers previously validated with individuals with unilateral above-knee amputation^18,22–25^. Different from previous studies, the two powered prostheses run similar but independent control algorithms, and do not communicate during operation. We compare the kinematics and kinetics of the powered prostheses to that of the prescribed microprocessor-controlled, passive prostheses and to normative data obtained from nonamputee datasets. By restoring natural ambulation in walking, and especially in stairs, powered prostheses may improve the mobility and quality of life of individuals with bilateral above-knee amputations.

## Results

### Bilateral gait patterns in walking, stair ascent, and stair descent

**Figure 1:**
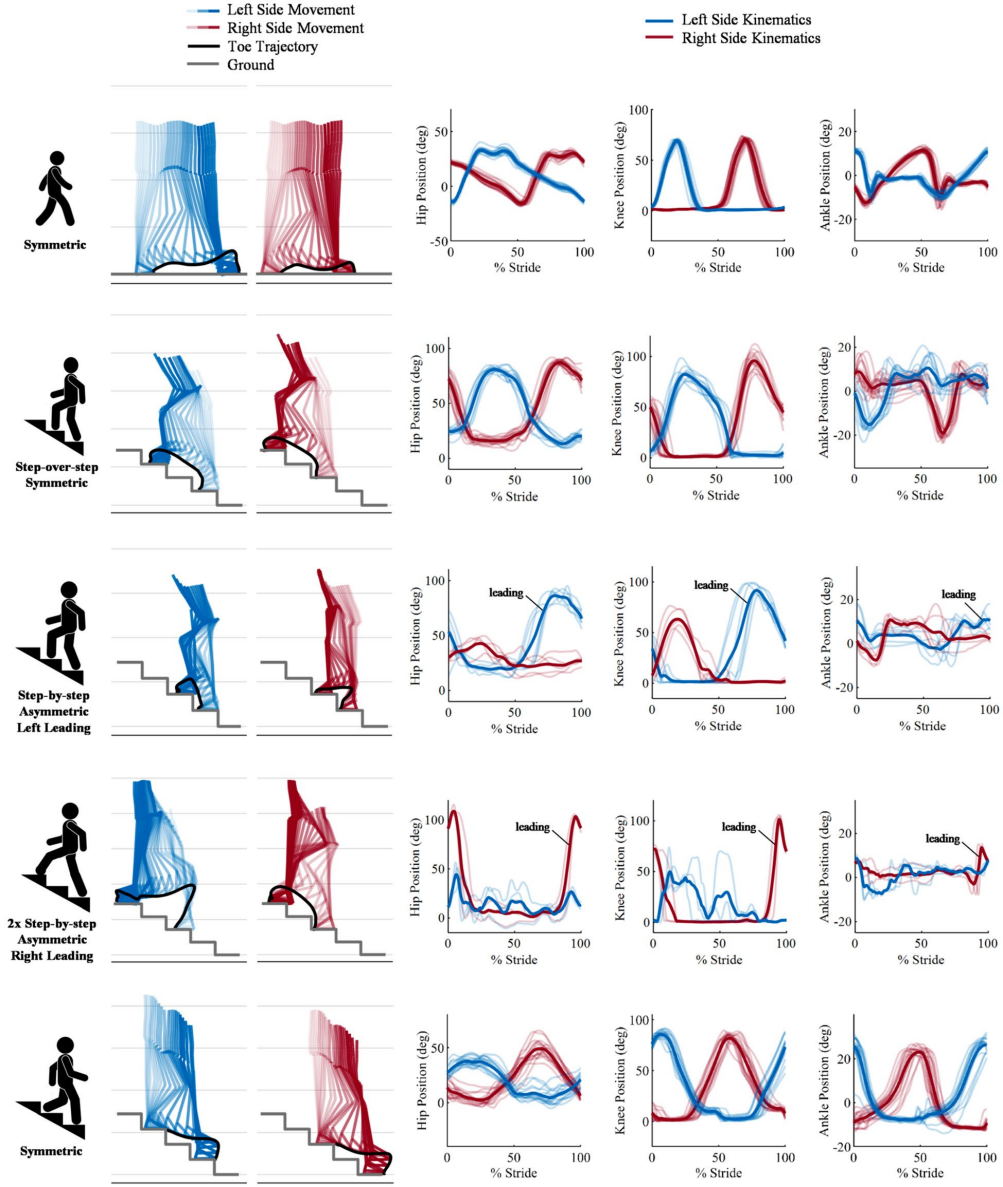
Cartesian space and joint kinematics for five ambulation activities of an individual with bilateral above-knee amputations. The cartesian space, sagittal plane movement of the participant wearing the powered prostheses are shown for the left side (blue) and the right side (red) with the lighter shade of color depicting the start of the movement and the darker shade of color depicting the end of the movement. The toe trajectory during the movement is represented by the solid black line. The level ground in walking or the stair configuration for stair ambulation is represented by the solid gray line. The hip, knee and ankle kinematic profiles (left to right) are shown with both the left side in blue and right side in red segmented for % Stride of the right leg for symmetric gait patterns, and % Stride of the leading leg for asymmetric gait patterns. All strides for each side are represented by a bolded average line. The activities represented are walking on level ground, stair ascent using symmetric step-over-step gait pattern, stair ascent using asymmetric step-by-step gait pattern with left side leading, stair ascent using asymmetric two-steps at a time step-by-step gait pattern with right side leading, and stair descent (top to bottom).

The participant walked on level-ground and climbed stairs with two lightweight powered knee and ankle prostheses without explicit synchronization between the devices. The analysis of the toe trajectory during walking shows that the powered prostheses allow for sufficient clearance through coordinated knee flexion and ankle dorsiflexion (Fig. 1, Supplementary Video 1), even though there are noticeable differences between the right, dominant side, and the left side. The observed differences in toe trajectories are particularly visible towards the end of swing, with the non-dominant, left side showing greater clearance right before heel strike (19.7 vs. 10.6 cm). Focusing on stair ambulation, we observe that the participant was able to ascend stairs using both a symmetric, step-over-step gait pattern and an asymmetric, step-by-step gait pattern, using the same stair ascent controller (Fig. 1). When using the step-over-step gait pattern, both the left side and the right side powered knee prostheses approached the step in a similarly flexed angle. Also, as the powered prostheses approach the step, both left side and right side ankle joints are dorsiflexed, allowing for the prosthetic feet to lay flat on the step, ready for the participant to put weight on the powered prostheses and climb the step. As expected from this symmetric step-over-step gait pattern, there are minimal differences between the toe trajectories of the left and right sides (peak vertical toe position of 51.7 vs. 52.9 cm). Using the same controller, the participant was able to ascend stairs with a step-by-step gait pattern, both one and two steps at a time (Fig. 1, Supplementary Video 2). When using an asymmetric, step-by-step gait pattern, the leading and trailing legs perform different movements, resulting in substantially different toe trajectories as well as the kinematics of the and hip, knee, and ankle joints between the left and right sides (Fig. 1). Despite the asymmetric gait pattern, the analysis of the toe trajectories show that the powered prostheses provide adequate clearance for both the left and right sides in all tested conditions. Not surprisingly, the kinematic analysis shows that both toe trajectories and joint kinematic profiles are different between climbing one step and two steps at a time. When climbing one step at a time, the leading leg shows a difference of 4.0 cm from the start to the horizontal peak of the toe trajectory vs. 13.7 cm with the trailing leg. When climbing two steps at a time, the leading leg shows a difference of 2.6 cm from the start to the horizontal peak of the toe trajectory while the trailing leg shows a difference of 24.1 cm. Using a dedicated controller, the participant was able to descend stairs with a natural, symmetric gait pattern. The left and right sides show less than 1% difference in horizontal peak toe positions (65.4 vs. 65.9 cm) when descending stairs. Notably, the participant was able to keep the prosthetic feet flat on the step during stance, which is not possible with prescribed passive prostheses. In stance, the concurrent ankle dorsiflexion and knee flexion enabled the participant to gently lower his body mass while slowing down the momentum (Fig. 1, Supplementary Video 3). In swing, the knee extends in preparation for the next step to be taken. The lightweight powered prostheses and related controllers enabled the individual with bilateral above-knee amputations to ambulate both on level-ground and on stairs with natural gait patterns.

### Kinematic and kinetic analysis for walking, stair ascent, and stair descent

The kinematic and kinetic analysis in walking shows that both the powered and passive prostheses can enable a bilateral above-knee amputee user to walk over level ground. However, only the powered prostheses can provide forward momentum by generating positive ankle power and show natural ankle plantarflexion at push-off—a critical factor for gait efficiency^27^. Our kinematic and kinetic analysis quantifies differences between powered and passive prostheses during walking by using three clinically relevant goals: *controlled weight acceptance, forward propulsion, and swing clearance* (Table 1). The statistical analysis performed on the walking metrics associated with these clinically relevant goals shows significant differences between the powered and passive prostheses, and between the passive and nonamputee reference (Table 1).

**Table 1:** Clinically relevant goals and metrics during walking for the powered and passive prostheses.

Walking			
Clinically relevant goal	Metric	Device	Avg [left/right]
Controlled weight acceptance	Hip flexion angle at heel strike (deg)^a,b,c^	Goal	19.5
		Powered	22.0 [20.9/23.0]
		Passive	23.3 [21.1/25.4]
	Peak knee flexion in early stance (deg)^a,b,c^	Goal	24.2
		Powered	1.2 [1.0/1.3]
		Passive	1.9 [1.8/2.0]
	Ankle plantarflexion angle in early stance (deg)^a,b,c^	Goal	− 3.2
		Powered	− 11.0 [− 9.3/− 12.8]
		Passive	− 4.4 [− 6.1/− 2.8]
Forward Propulsion	Knee flexion torque (Nm)	Goal	− 15.4
		Powered	− 15.5 [− 11.9/− 19.1]
		Passive	–
	Peak ankle plantarflexion (deg)^a,b,c^	Goal	− 15.8
		Powered	− 9.5 [− 8.2/− 10.8]
		Passive	− 1.2 [− 2.4/− 0.1]
	Ankle push-off power (W)^b^	Goal	182.6
		Power	93.3 [88.4/98.3]
		Passive	–
Swing Clearance	Peak knee flexion (deg)^a,b,c^	Goal	63.5
		Powered	71.0 [69.9/72.0]
		Passive	60.4 [62.3/58.6]

^a^Statistically significant difference between powered and passive prostheses.

^b^Statistically significant difference between powered prostheses and nonamputee reference.

^c^Statistically significant difference between passive prostheses and nonamputee reference.

**Figure 2:**
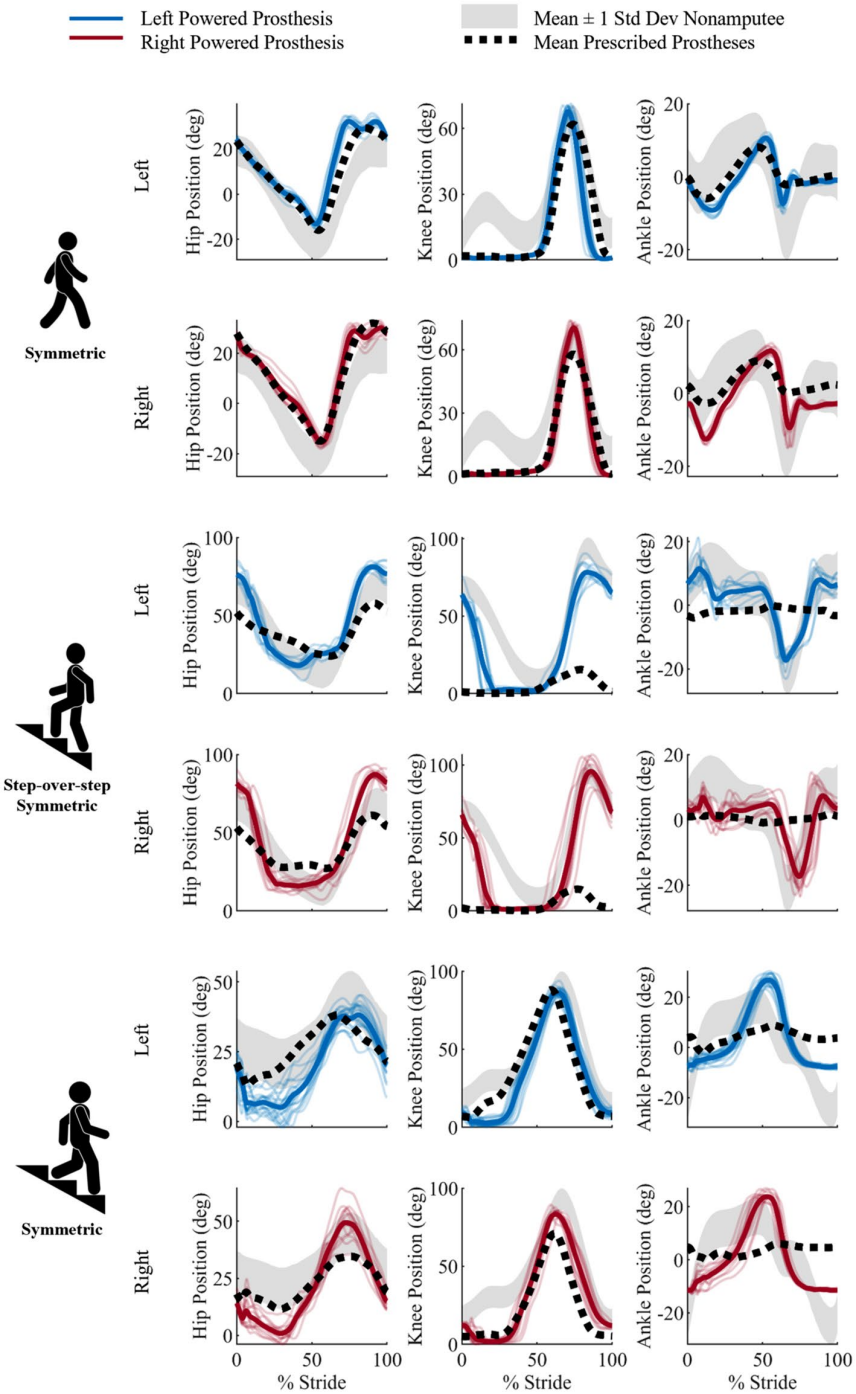
Kinematic profiles of the hip, knee and ankle joints during all ambulation activities. Specifically, the hip, knee, and ankle kinematics profiles (left to right) are shown for both the left side (top row) and right side (bottom row) for each ambulation activity. The ambulation activities are walking, stair ascent using the symmetrical step-over-step gait pattern, and stair descent (top to bottom). The solid blue lines represent the left side and the solid red lines represent the right side of the powered prostheses kinematic profiles with all strides represented by a bolded average line. The black dotted line is the average of the prescribed passive prostheses kinematics profiles. The shaded gray region is the standard deviation of the nonamputee reference data^26^.

**Figure 3:**
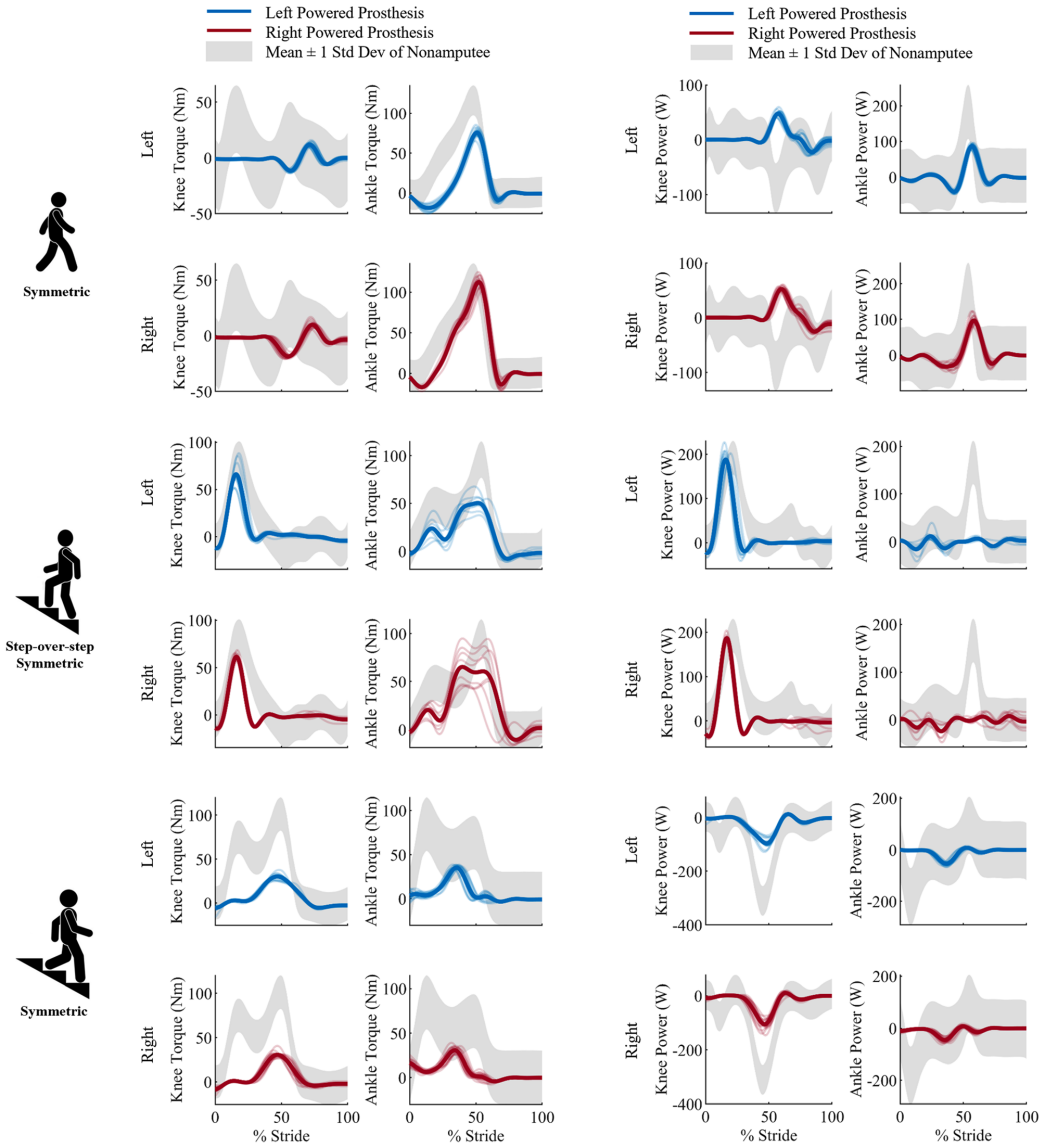
Kinetic profiles of the knee and ankle joints during all ambulation activities. Specifically, the knee and ankle torque profiles and the knee and ankle power profiles (left to right) are shown for both the left side in blue (top row) and right side in red (bottom row) for each ambulation activity. The ambulation activities are walking, stair ascent using the symmetrical step-over-step gait pattern, and stair descent (top to bottom). The powered prostheses torque and power profiles are shown with all strides represented under a bolded average line. The shaded gray region is the standard deviation of the nonamputee reference data^26^.

*Controlled weight acceptance* is associated with kinematic variables such as hip flexion at heel strike and peak knee and ankle angles in early stance. The powered prostheses do not provide knee flexion in early stance and instead remain fully extended against their mechanical end-stops (Fig. 2). As a result, there is no active knee torque or power in early stance from the powered prostheses (Fig. 3) compared to the nonamputee reference which shows an early stance knee extension torque to contribute to the goal of controlled weight acceptance. Also, in early stance, the powered prostheses show 244% greater early stance ankle plantarflexion angle than the target value. In comparison, the passive prostheses achieve 140% of the target value (Table 1).

*Forward propulsion* is measured by the knee flexion torque, peak ankle plantarflexion in late stance, and ankle push-off power. The powered prostheses fundamentally accomplish the goal of late stance peak ankle plantarflexion, although with the powered prostheses the late stance peak ankle plantarflexion angle is somewhat lower than the target value (39% lower). In contrast, the prescribed passive prostheses do not provide any meaningful plantarflexion in late stance (92% lower), failing to satisfy this reference metric. Finally, the powered prostheses partly achieve the target value for the ankle push off power (49% lower, Table 1).

*Swing clearance* is another clinically relevant goal for walking. Peak knee flexion is a critical factor in achieving this goal. The powered prostheses exceed the target by 12% while the passive prostheses undershoot this target by 5%. Swing clearance is achieved by the powered prostheses providing the necessary torque and power at the knee using closed-loop position control (Fig. 3).

The analysis of stair ascent shows that the powered prostheses can achieve close to the normative kinematics and kinetics, which is not possible with the passive prostheses. Also, our analysis shows that the unique ability of powered prostheses to generate knee extension torque in stance is a major factor in achieving this result. Our kinematic and kinetic analysis quantifies differences between the powered and passive prostheses using three clinically relevant goals: *vertical propulsion*, *swing clearance*, *and minimal upper limb support* (Table 2). For all the kinematic metrics associated with these clinically relevant goals, there is a statistically significant difference between the powered and passive prostheses and between the passive prostheses and nonamputee reference (Table 2).

**Table 2:** Clinically relevant goals and metrics during stair ascent for the powered and passive prostheses.

Stair ascent			
Clinically relevant goal	Metric	Device	Avg [left/right]
Vertical propulsion	Hip flexion angle at foot contact (deg)^a,b,c^	Goal	66.5
		Powered	79.6 [76.8/82.4]
		Passive	53.8 [53.1/54.5]
	Knee flexion angle at foot contact (deg)^a,c^	Goal	69.1
		Powered	67.4 [66.1/68.7]
		Passive	1.7 [1.4/2.1]
	Peak knee extension torque (Nm)^b^	Goal	82.0
		Powered	65.1 [68.0/62.2]
		Passive	–
	Peak knee extension power (W)	Goal	192.1
		Powered	193.8 [199.1/188.4]
		Passive	–
	Peak ankle plantarflexion torque (Nm)^b^	Goal	95.9
		Powered	59.8 [51.6/67.9]
		Passive	–
Swing clearance	Peak knee flexion angle (deg)^a,c^	Goal	93.8
		Powered	88.9 [80.6/97.2]
		Passive	15.6 [16.2/14.9]
	Peak ankle plantarflexion angle (deg)^a,b,c^	Goal	− 20.8
		Powered	− 19.0 [− 18.1/− 19.9]
		Passive	− 2.7 [− 3.8/− 1.6]
Minimal upper limb support	Peak vertical GRF (%BW)^b^	Goal	107
		Powered	81 [63/99]
		Passive	–

^a^Statistically significant difference between powered and passive prostheses.

^b^Statistically significant difference between powered prostheses and nonamputee reference.

^c^Statistically significant difference between passive prostheses and nonamputee reference.

*Vertical propulsion* is measured by the hip and knee angles at foot contact, peak knee extension torque and power during stance, and the ankle plantarflexion torque. The powered prostheses satisfy the target goal for knee flexion angle at foot contact, achieving 98% of the target value. In contrast, the passive prostheses miss the target, achieving only 2% of the target value (Table 2). The target peak knee extension torque is another key metric of vertical propulsion. The powered prostheses show a peak knee extension torque which is 79% of the target value (Fig. 2). Moreover, the powered prostheses achieve 101% of the target knee extension power value (Table 2), which is another metric associated with vertical propulsion. In contrast, the powered prostheses only achieve 62% of the target value for the peak ankle plantarflexion torque (Table 2).

*Swing clearance* is another clinically relevant goal in stair ascent and the peaks of the knee flexion angle and the ankle plantarflexion angle are critical factors in achieving this goal. The powered prostheses fundamentally achieve the target for peak knee flexion angle by providing 95% of the target knee flexion angle. The passive prostheses do not accomplish this goal, achieving only 17% of the target knee flexion value. The powered prostheses essentially meet the target for the peak ankle plantarflexion angle, achieving 91% of the target value. In contrast, the passive prostheses do not accomplish this goal, as they only achieve 13% of the target value of peak ankle plantarflexion angle.

*Minimal upper-limb support* is a clinically relevant goal for stair ascent. This goal is assessed by measuring the peak ground reaction force normalized by body weight. The results show that there is a significant difference between the left and the right sides. The right, dominant side fundamentally accomplishes the goal, achieving 93% of the target value. In contrast, the left side only shows 59% of the target value.

The analysis of stair descent shows that the powered prostheses provide lower knee and ankle torque than the able-bodied reference, although this level of torque is sufficient for the participant to descend stairs safely. Notably, only the powered prostheses can imitate the range of motion of the biological ankle. The extended ankle range of motion in the powered prosthesis allows for the foot to be placed flat on the step during stance (Fig. 1), providing a more natural and stable stair-descent motion. Our kinematic and kinetic analysis assess stair descent ambulation by using two main clinically relevant goals, *controlled yielding* and *minimal upper limb support* (Table 3). The statistical analysis shows significant difference for all kinematic metrics between the powered and passive prostheses, and between the passive prostheses and nonamputee reference. Moreover, the statistical analysis shows significant differences between the powered prostheses and the nonamputee references, for several kinematic and kinetic metrics (Table 3).

**Table 3:** Clinically relevant goals and metrics during stair descent for the powered and passive prostheses.

Stair descent			
Clinically relevant goal	Metric	Device	Avg [left/right]
Controlled yielding	Hip flexion at foot contact (deg)^a,b,c^	Goal	27.9
		Powered	10.3 [13.4/7.1]
		Passive	16.1 [16.9/15.4]
	Ankle plantarflexion at foot contact (deg)^a,b,c^	Goal	− 21.2
		Powered	− 8.7 [− 6.9/− 10.5]
		Passive	2.6 [3.4/1.8]
	Peak ankle dorsiflexion (deg)^a,b,c^	Goal	16.4
		Powered	25.8 [27.0/24.5]
		Passive	8.5 [9.4/7.5]
	Peak knee extension torque (Nm)^b^	Goal	101.1
		Powered	30.6 [30.2/31.0]
		Passive	–
	Negative Peak Knee Power (W) ^b^	Goal	− 310.6
		Powered	− 102.5 [− 96.2/− 108.9]
		Passive	–
	Peak ankle plantarflexion torque (Nm)^b^	Goal	84.5
		Powered	34.4 [36.5/32.2]
		Passive	–
Minimal upper limb support	Peak vertical GRF (%BW)^b^	Goal	126
		Powered	83 [84/81]
		Passive	–

^a^Statistically significant difference between powered and passive prostheses.

^b^Statistically significant difference between powered prostheses and nonamputee reference.

^c^Statistically significant difference between passive prostheses and nonamputee reference.

*Controlled yielding* is among the most important clinically relevant goals for stair descent and is assessed by six kinematic, and kinetic metrics across the hip, knee and ankle joints. The peak ankle dorsiflexion angle in stance is critical for controlled yielding. The powered prostheses exceed the target peak ankle dorsiflexion angle, achieving 157% of the target value (Fig. 2). In contrast, the passive prostheses do not satisfy this goal, and the peak ankle dorsiflexion angle is only 52% of the target value. Another metric used to quantify controlled yielding is the peak knee extension torque during stance. The powered prostheses peak knee extension torque is 30% of the target value. The knee extension torque results in a negative peak power, which is another metric for controlled yielding. The powered prostheses have a peak knee power absorption of 67% of the target negative peak power (Fig. 3). Another metric used to quantify *controlled yielding* is peak ankle plantarflexion torque. The powered prostheses achieve 41% of the target peak ankle plantarflexion torque. *Minimal upper limb support* is another clinically relevant goal for stair descent and is also measured by the peak ground reaction force. The powered prostheses achieve 66% of the nonamputee reference for peak ground reaction force, with minimal differences between the left and the right sides (< 4%).

## Discussion

Bilateral above-knee amputation severely limits ambulation ability and quality of life^1,3,4^. Powered prostheses have the potential to address this problem by closely replicating the biomechanical functions of the missing legs during virtually all ambulation activities^17,18,23,24,28^. Two previous case studies^15,16^ have shown that powered knee and ankle prostheses can enable an individual with bilateral above-knee amputation to walk on level ground. In this case study, we show that powered prostheses can enable the participant to climb stairs both with a symmetric, step-over-step gait pattern and an asymmetric step-by-step gait pattern, one or two steps at a time, which is not possible with conventional passive prostheses. Also, the participant could descend stairs with a natural step-over-step gait pattern while keeping the whole foot on the step, which, again, is not possible with conventional passive prostheses. To the best of our knowledge, this case study shows, for the first time, that powered knee and ankle prostheses can restore natural ambulation on stairs in one individual with bilateral above-knee amputations. Moreover, we show that powered prostheses can replicate the key biomechanical functions of the missing biological ankles during walking by generating positive power and aiding gait propulsion. This case study is a first step towards enhancing mobility and quality of life for individuals with bilateral above-knee amputations with powered knee and ankle prostheses.

During walking, the biological knee joint provides early stance knee flexion to allow for controlled weight acceptance after heel strike. Although both passive and powered prostheses can theoretically provide stance knee flexion, individuals with above-knee amputations rarely use this function. Passive-prostheses users tend to exaggerate hip extension at heel strike^9^ to ensure the prosthetic knee stays fully extended against the end stop as they load the prostheses. This compensatory hip movement prevents stance knee flexion in knee prostheses. In agreement with previous studies, we see a lack of stance knee flexion with both the powered and passive prostheses, most likely a result of the habitual compensatory movement. However, we also see that the powered ankle prostheses deliver a larger plantarflexion angle compared to the nonamputee reference. In contrast, passive ankle prostheses do not provide extended ankle plantarflexion. These results suggest that the powered ankle prostheses can compensate for the lack of early stance knee flexion, providing controlled weight acceptance after heel strike.

In late stance walking, the biological ankle provides push-off power for forward propulsion. Our results show that the powered ankle prostheses can replicate this biomechanical function. However, the ankle prostheses push-off power is lower than the nonamputee reference, whereas the peak of the ankle plantarflexion torque is similar to the nonamputee reference. Thus, the difference in push-off power seems to be due to a mismatch between the joint velocities of the powered prosthetic ankle and the biological ankle. Interestingly, the push-off power is stronger on the right, dominant side than the left, non-dominant side. Conversely, the passive prostheses show substantial differences in late stance ankle kinematics and kinetics compared to the nonamputee reference, showing they cannot provide ankle push-off for forward propulsion. Thus, powered ankle prostheses can more closely approximate the biomechanical functions of the biological ankles.

In level-ground swing, the knee flexes and extends to provide timely foot placement while ensuring appropriate swing clearance. Swing clearance is achieved by the powered prostheses using ankle and knee kinematic profiles similar to the nonamputee reference. The passive prostheses also show similar knee kinematic profile to the nonamputee reference. However, the key benefit of the powered prostheses in swing clearance is the active control of the joint movements, which is reflected in the kinetic profiles during swing phase. When an amputee walks with the passive prostheses, the swing clearance is achieved by the transfer of user-generated momentum from the hip joint to the prostheses. As a result, powered prostheses can provide added ankle plantarflexion for controlled weight acceptance, provide push off power at the ankle for forward propulsion, and generate movement for swing clearance. Thus, our results suggest that powered prostheses can restore a more natural walking pattern than passive prostheses.

During stair ascent, when the foot is in contact with the step, the biological knee and ankle joints generate extension torque and positive power for vertical propulsion. Passive prostheses lack the ability to provide positive power generation for vertical propulsion. As a result, the user performs unnatural movements for stair ascent that involves significant upper limb support. Specifically, in stance, the user places his prosthetic foot on the edge of the step with the knee fully extended. Once the foot is on the step, he needs to exert unnatural hip extension torque to keep the prosthetic knee fully extended against the end-stop while he pulls himself up the step using his upper body strength. In contrast, powered prostheses can actively generate extension torque to provide vertical propulsion. This case study shows that powered prostheses can replicate nonamputee biomechanics for knee extension torque and power during stance. We also show that the biologically accurate knee extension torque and power enable the participant to climb stairs using minimal upper-limb support (Fig. 3, Table 2). Interestingly, the upper-limb support was lower for the right, dominant side than the left, non-dominant side. This result may be the consequence of differences in residual-hip strength. Another possible explanation is that the participant felt more confident when leading with his right, dominant side. Thus, this case study suggests that climbing steps with powered prostheses is significantly less challenging than with passive prostheses.

During stair ascent, when the foot is off the ground, the biological hip, knee, and ankle joints coordinate their movements to achieve both proper clearance and correct placement of the foot on the step. Because passive prostheses cannot actively control knee movements, the prosthetic knee does not flex in swing as needed to clear the step. To compensate for the lack of knee flexion, the participant needs to circumduct his hip, placing the prosthetic foot on the edge of the step with the knee fully extended. This requires additional compensatory movements with the upper and lower body. In contrast, the powered prostheses actively control the knee and ankle joints to generate biologically accurate movements, enabling the participant to clear the steps while achieving a natural foot placement on the step. Moreover, we show that using a stair ascent controller based on the natural hip-knee-ankle synergy enables the user to achieve controlled swing clearance and proper foot placement when climbing stairs one or two steps at a time, step-by-step or step-over-step. Thus, this case study suggests that powered prostheses enable ascending stairs naturally, which is not possible with conventional passive prostheses.

During stair descent, the biological knee and ankle joints provide negative power throughout a wide range of motion to control the movement of the center of mass with minimal upper limb support. This yielding function is achieved by biological legs through the simultaneous generation of knee extension torque and ankle plantarflexion torque. Passive knee prostheses can control the knee extension resistance, providing negative power as needed to control the downward movement of the body mass in stair descent. However, most passive ankle prostheses cannot actively control the plantarflexion resistance and do not have the range of motion necessary to restore natural stair descent. As a result, the participant needs to place his foot on the edge of the step, pivoting on the prosthetic foot as he rolls his body forward while the knee flexes. This unnatural stair descent strategy is inherently risky because the prosthetic foot can easily slip off the edge of the step, causing the user to fall. The powered prostheses address this critical issue by enabling the user to place the whole prosthetic foot on the step.

Our results show that the powered prostheses can imitate the natural yielding function during stair descent. However, the peaks of the knee and ankle torque are much lower than the target references extracted from nonamputee biomechanics (Fig. 3, Table 3). These differences are not due to a fundamental limitation of the powered prostheses or the controller in use but rather to the specific prostheses tuning used for the experiments. Following pilot tests, we reduced the resistive torque provided by the powered prostheses. The participant found it too challenging to initiate knee flexion during early stance when the powered prostheses were tuned to replicate the physiological torque levels. We speculate that the subjective preference for low prostheses yielding could be due to a lack of confidence with the powered prostheses. This speculation is drawn from the suggestion of upper limb support by the participant, therefore, not loading his full body weight on the prostheses. Thus, this case study suggests that powered prostheses provide safer and more natural stair descent functionality than passive prosthesis. However, there are visible deviations from the biological joint kinetics that need to be further explored.

Although this case study shows promising results, it does have limitations. The participant had very limited training and experience with the powered prostheses compared to his passive prescribed prostheses. Based on previous studies^29^, we expect that confidence and performance will increase with further training. The kinetic data was obtained from the sensors embedded in the prostheses rather than inverse dynamic analysis. Although this kinetic analysis is commonly used in the powered prosthetics community, it is less accurate than motion capture and it cannot capture the effect of the mechanical end-stops. The research powered prostheses used in this study are about half the weight of other powered prostheses used in previous studies with individuals with bilateral amputations^15,16^ while being able to generate the same or greater torque, power, and range of motion. It is likely that the hardware specifications of powered prostheses such as those mentioned above have substantial impact on the ability of an individual with bilateral amputation to ambulate. The results of this study may not generalize to the commercially available powered prostheses, the Ossur Power Knee^30^ and Ottobock Empower Ankle^31^, which are heavier, have lower torque limits, and much smaller range of motion compared to the powered prostheses used in this study. Finally, the participant recruited for this case study is a full community ambulator, therefore performing at a very high level compared to an average individual with bilateral above-knee amputations. Further experiments are necessary to understand the outcome of powered prostheses in the broader bilateral above-knee amputee population. Despite its limitations, this case study shows, for the first time, that powered knee and ankle prostheses have the potential to restore natural ambulation on level ground and stairs for individuals with bilateral above-knee amputations.

## Methods

### Experimental protocol and data processing

We recruited one individual with bilateral above-knee amputations to participate in this case study. Prior to participation, the individual provided written informed consent, including consent for use of photos and videos of the experiment for dissemination purposes. All study protocols were approved by the University of Utah Institutional Review Board and were performed in accordance with the approved protocol. The participant is a 46-year-old man, who has had his above-knee amputations for 7 years and is a full-community ambulator. The participant has used a pair of Ottobock C-Leg Genium knees in combination with Ottobock Triton feet for 4 years. With his prescribed prostheses, the participant weighs 76 kg and is 1.76 m tall.

A certified prosthetist performed the fitting and alignment of the powered prostheses at the beginning of each experimental session. For the initial training sessions, the powered prostheses were built to match the height of the participant’s prescribed prostheses. After familiarization with the powered prostheses, the build height was adjusted to match the participant’s anthropometrics following Dempster’s assumptions for anatomical segment lengths^32^. Specifically, the powered prostheses shank-segment length, measured from the knee joint center of rotation to the ankle joint center of rotation, was fitted to proportionally match the participants thigh segment length. The participants thigh segment length was measured from the hip joint center of rotation to the prosthetic knee joint center of rotation when the prostheses were fitted to the distal end of the participant’s sockets. With the powered prostheses fitted to match the participant’s anthropometrics, the participant is 1.83 m tall.

Prior to participation in this study, the participant had no previous experience with powered prostheses. Training with the powered prostheses consisted of five sessions spread out over the course of 3 months. Each training session lasted between 2 and 3 h and were at least 1 week apart. For each training session, the participant started by donning an IMU-based motion capture system (MTw Awinda Xsens, Enschede, Netherlands) and performing the required system calibration. The calibration required the participant to stand still, walk 4 m forward, turn around and return to the initial starting pose. After successful calibration, the participant performed ambulation activities specific to the session with his prescribed passive prostheses. If the goal for the day was to train on walking, the participant performed roughly 10 laps on a 10-m walkway. If the goal for the day was to train on stairs, then the participant performed roughly 5 stair ascents and descents on a staircase with 4 ADA-compliant steps. After finishing the protocol with the prescribed passive prostheses, the participant donned the powered prostheses. The participant took a few walking steps with the powered prostheses to acclimatize. Concurrently the certified prosthetist made small adjustments to the fitting and alignment. At this point, the participant was still wearing the motion capture system, and he repeated the motion capture calibration now with the powered prostheses. Then, the participant began training of the planned ambulation activity for the remainder of the session.

When a new ambulation activity was introduced, the tuning and calibration of the powered prostheses were initially set to replicate nonamputee biomechanics following previous validation of each ambulation controller. As the participant trained, small, incremental changes of the tuning were adjusted to the participant’s preference (Table 4). For the walking controller^22^, the participant preferred tuning with no early stance knee flexion, different from nonamputee biomechanics. For stair ascent^18^, the participant placed one leg at rest on the step, and the knee position and thigh position were measured. These measurements were used for participant specific tuning of the knee-thigh position relationship, ${k}_{1}^{knee}$ and ${k}_{1}^{ankle}$, for the adaptive stair ascent controller. The tuning for stair ascent was consistent across all stair ascent gait patterns performed by the participant. For stair descent^17,19^, the participant preferred one third of the peak knee extension torque during stance compared to the nonamputee biomechanics. The participant found it challenging to overcome the nonamputee biomechanics level of resistance and achieve knee flexion.

**Table 4:** Ambulation activity parameters tuned to participant preferences.

Ambulation activity	Parameter	Value
Walking^22^	Body mass	76 kg
	Swing flexion duration	0.12 s
	Swing extension duration	0.32 s
	Peak knee flexion swing	60 deg
Stair ascent^18^	Knee position at max torque % of knee position heel strike	90%
	Max torque % nonamputee references	100%
	${k}_{1}^{knee}$	1.67 deg/deg
	${k}_{1}^{ankle}$	0.75 deg/deg
	${k}_{4}^{0, knee}$	2000 1/g
	${k}_{4}^{0, ankle}$	1500 1/g
Stair descent^17,19^	Knee damping stance	0.1 Nm s/deg
	Ankle damping stance	0.4 Nm s/deg
	End knee angle swing	10 deg

Data collection took place over 3 days in three separate weeks. One day of data collection was for walking, 1 day was for step-by-step stair ascent and stair descent, and 1 day was for step-over-step stair ascent. The participant followed a protocol similar to the training sessions. The protocol first started with donning and calibrating the motion capture system with the prescribed prostheses. Then, the participant performed the selected ambulation activity for the session with the prescribed prostheses. Next, the participant donned the powered prostheses, fitted and aligned by a certified prosthetist. The participant took a few steps to acclimatize with the powered prostheses. Concurrently, the certified prosthetist made final adjustments. Then, the participant recalibrated the motion capture system with the powered prostheses. Finally, the remainder of the session the participant performed the selected ambulation activity with the powered prostheses.

All kinematic data was obtained from the motion capture system. The motion capture data collected with the passive and powered prostheses were analyzed using the Xsens offline algorithms for joint kinematics. Specifically, the motion capture data was processed using the ‘*single-level*’ option for level-ground walking, and the ‘*multi-level*’ option for stair ascent and descent as these activities require changes in elevation. As commonly done for powered prostheses in the field^14,17^, we estimated the powered prostheses joint torque and power through the measurement of the motor currents. Specifically, the powered prostheses joint torque is obtained offline using a dynamic model of the actuation system that includes Coulomb friction, viscous damping, and the motor/transmission inertia. Similar to our previous work^17,28^, the parameters of the dynamic model are estimated experimentally, and the model was validated using an external load cell in a separate study. We calculated the power profiles for the powered prostheses offline by multiplying joint torque by joint velocity, which is also calculated offline based on the joint angle measurements provided by the encoders embedded in the prostheses. A zero-phase, fourth-order Butterworth filter with a cutoff frequency of 2.5 Hz was applied to all kinematic and kinetic data. Joint kinematics, kinetics, and power were segmented from heel strike to heel strike using MATLAB (Mathworks, Natick, MA). The average trajectories for each joint and activities were calculated offline in MATLAB.

The key metrics related to clinically relevant goals were extracted from the kinematics of both the powered and passive protheses, and from the kinetics of the powered prostheses. Each metric was averaged across strides for the left side, right side, and between both sides. The peak vertical GRF metric was calculated from the measured ground reaction force by the powered prostheses and the participants body weight. A paired T-Test was performed on each metric of the clinically relevant goals, comparing the nonamputee vs. powered prostheses, nonamputee vs. passive prostheses, and the powered vs. passive prostheses. The threshold for statistical significance was p < 0.05. The significance of each test was adjusted with the Bonferroni correction, accounting for the use of three simultaneous statistical comparisons.

### Powered prosthesis and ambulation controllers

**Figure 4:**
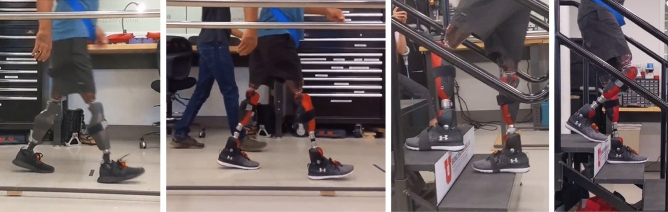
The passive and powered prostheses donned by the participant. From left to right, the images show the participant walking with his prescribed prostheses, walking with the powered prostheses, ascending stairs step-over-step with the powered prostheses, and descending stairs with the powered prostheses. For all conditions the participant was wearing Xsens Motion Tracking IMUS on the prostheses.

For this study, we used two replicas of the Utah Bionic Leg, a self-contained, battery-operated, lightweight robotic leg prosthesis. The Utah Bionic Leg (Fig. 4) has dedicated knee and ankle/foot modules capable of providing biomechanically accurate torque during ambulation. To achieve high torque in a compact and lightweight device, the powered knee modules use a novel torque-sensitive actuator that combines the benefits of variable transmission^17^ with that of series-elastic actuators^33^. The powered ankle/foot modules have two articulated joints, the ankle and the toe, which are powered by a single actuator using a compliant underactuated mechanism^34^. A custom instrumented pyramid adapter is located at the top of the ankle modules to estimate the vertical ground reaction force and the torque in the sagittal plane^35^. The knee and ankle/foot modules are mechanically connected with a standard prosthetic pylon, which is cut to size for each user, like in commercially available prostheses. A digital communication line passes through the pylon enabling the knee and ankle modules to synchronize their movements during ambulation. Combined, the powered knee and ankle/foot modules weigh only 3.2 kg including batteries and protective covers, this weight is about half the that of the powered prostheses used in previous studies with individuals with bilateral above-knee amputations^15,16^.

**Figure 5:**
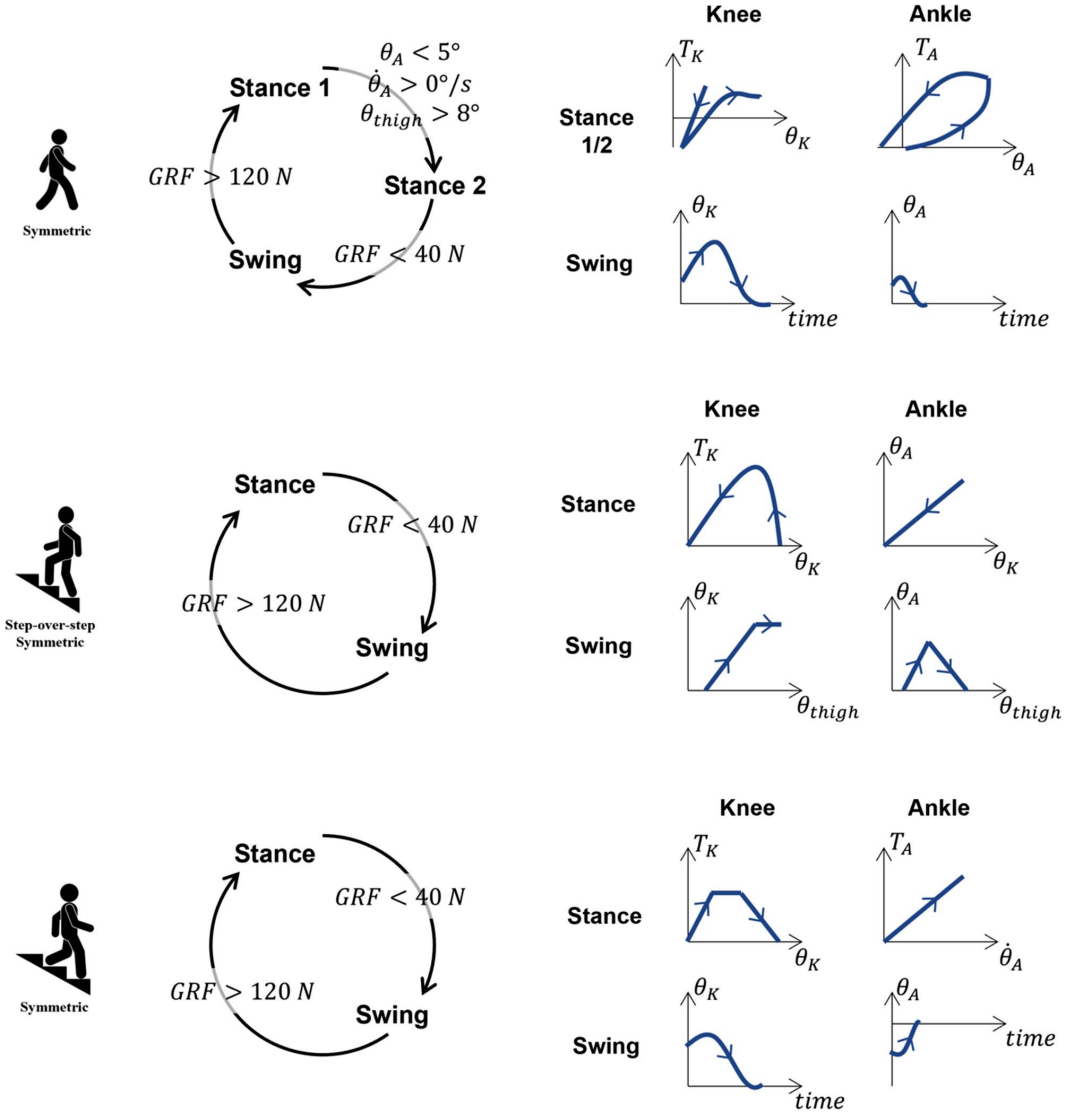
Control architecure of the Utah Bionic Leg. The schematic representation of the hierarchical architecture for the high and mid-level control implemented on the Utah Bionic Leg. Dedicated controllers are used for walking, stair ascent, and stair descent (top to bottom) using transition conditions between defined states, and the joint profiles for each state (left to right). Symbols $T$ and $\theta$ denote joint torque and angle. Subscripts K and A denote knee and ankle joints.

The Utah Bionic Leg uses a hierarchical control system enabling different ambulation modes. The desired ambulation mode is selected manually by the experimenter through a graphical interface. The high-level controller uses a finite-state machine with a specific number of states and transition rules for each ambulation mode (Fig. 5). The mid-level controller uses different control algorithms for each state to generate either a desired joint torque or joint position. The low-level controller translates the desired joint torque or position into current commands for the knee and ankle/foot motors. For both the knee and ankle/foot modules, the position controller is based on a simple proportional-derivative (PD) compensator with additional feed-forward terms for gravity and friction compensation. The desired torque is translated into motor current commands using specific algorithms for the knee and ankle modules. This hierarchical control strategy has been shown to enable individuals with a unilateral above-knee amputation to walk^17,25^, ascend, and descend stairs^18,19^.

In walking, we use three states: Stance 1 (from heel strike to mid stance), Stance 2 (mid stance to toe off), and Swing (toe off to heel strike). In Stance 1 and Stance 2, the desired torques for the knee and ankle joints are defined based on the quasi-stiffness profiles of the biological leg^22,28^. In Swing, we use a minimum-jerk controller to generate biomimetic prosthesis trajectories for the ankle and the knee while maximizing smoothness^36,37^. In stair ascent, we use only two states: Stance and Swing. In Stance, we define the desired knee torque using a bell-shaped profile inspired by nonamputee data^26^. Also, in Stance, we change the desired angle of the ankle joint linearly with the knee angle, causing the ankle joint to move from a dorsiflexed angle to a neutral angle as the knee extends during stance. In Swing, the desired knee and ankle angles depend on the thigh orientation (i.e., residual limb) so that the knee flexes and the ankle dorsi-flexes proportionally to the thigh flexion. In stair descent, we use two states: Stance and Swing. In Stance, the desired knee torque changes as a function of the knee angle, following a trapezoidal torque profile (Fig. 5). Moreover, the desired ankle torque is proportional to the ankle velocity, behaving as a virtual damping. In Swing*,* the desired angles of the knee and the ankle joints are defined using a minimum-jerk profile, so that the knee and the ankle joint gently return to a neutral position enabling the participant to take the subsequent step. More details on the ambulation controllers can be found in our previous work^17–19,25^.

## Supplementary Information

Supplementary Video 1.

Supplementary Video 2.

Supplementary Video 3.

## Data Availability

The datasets used and/or analyzed during the current study are available from the corresponding author (sarah.hood@utah.edu) upon reasonable request.
